# ­Glial and stem cell expression of murine Fibroblast Growth Factor Receptor 1 in the embryonic and perinatal nervous system

**DOI:** 10.7717/peerj.3519

**Published:** 2017-06-29

**Authors:** Jantzen C. Collette, Lisha Choubey, Karen Müller Smith

**Affiliations:** Department of Biology, University of Louisiana at Lafayette, Lafayette, LA, United States of America

**Keywords:** Brain development, Stem cells, Radial glia, Ventricular zone, Hippocampus, Cortex, Corpus callosum, Indusium griseum, Prefrontal cortex, Bergmann glia

## Abstract

**Background:**

Fibroblast growth factors (FGFs) and their receptors (FGFRs) are involved in the development and function of multiple organs and organ systems, including the central nervous system (CNS). FGF signaling via FGFR1, one of the three FGFRs expressed in the CNS, stimulates proliferation of stem cells during prenatal and postnatal neurogenesis and participates in regulating cell-type ratios in many developing regions of the brain. Anomalies in FGFR1 signaling have been implicated in certain neuropsychiatric disorders. *Fgfr1* expression has been shown, via *in situ* hybridization, to vary spatially and temporally throughout embryonic and postnatal development of the brain. However, *in situ* hybridization lacks sufficient resolution to identify which cell-types directly participate in FGF signaling. Furthermore, because antibodies raised against FGFR1 commonly cross-react with other members of the FGFR family, immunocytochemistry is not alone sufficient to accurately document *Fgfr1* expression. Here, we elucidate the identity of *Fgfr1* expressing cells in both the embryonic and perinatal mouse brain.

**Methods:**

To do this, we utilized a *tgFGFR1-EGFPGP338Gsat* BAC line (*tgFgfr1-EGFP+*) obtained from the GENSAT project. The *tgFgfr1-EGFP+* line expresses EGFP under the control of a Fgfr1 promoter, thereby causing cells endogenously expressing *Fgfr1* to also present a positive GFP signal. Through simple immunostaining using GFP antibodies and cell-type specific antibodies, we were able to accurately determine the cell-type of *Fgfr1* expressing cells.

**Results:**

This technique revealed *Fgfr1* expression in proliferative zones containing BLBP+ radial glial stem cells, such as the cortical and hippocampal ventricular zones, and cerebellar anlage of E14.5 mice, in addition to DCX+ neuroblasts. Furthermore, our data reveal *Fgfr1* expression in proliferative zones containing BLBP+ cells of the anterior midline, hippocampus, cortex, hypothalamus, and cerebellum of P0.5 mice, in addition to the early-formed GFAP+ astrocytes of the anterior midline.

**Discussion:**

Understanding when during development and where *Fgfr1* is expressed is critical to improving our understanding of its function during neurodevelopment as well as in the mature CNS. This information may one day provide an avenue of discovery towards understanding the involvement of aberrant FGF signaling in neuropsychiatric disorders.

## Introduction

Fibroblast growth factors (FGFs) and fibroblast growth factor receptors (FGFRs) have a multitude of functions during embryonic development as well as in adult organisms. For example, FGF signaling participates in mesodermal patterning, organogenesis, angiogenesis, wound repair, and skeletal development and homeostasis. In addition, FGF signaling helps to regulate cellular proliferation, cell migration, cellular differentiation, and survival (Su, Jin & Chen, 2014; Rash et al., 2011; Turner & Grose, 2010; Li, Zhang & Kirsner, 2003; Wiedemann & Trueb, 2000; Kimelman & Kirschner, 1987). FGFs belong to a 22-member family of growth factors, most of which mediate their diverse regulatory functions by binding to and activating one of four different receptor tyrosine kinases designated FGFR1, FGFR2, FGFR3, and FGFR4, respectively (Eswarakumar, Lax & Schlessinger, 2005; Givol & Yayon, 1992; Lee et al., 1989). Canonical FGFR signaling is initiated by the binding of two FGF ligands to a pair of FGFR receptors, followed by autophosphorylation of the receptor dimer complex and subsequent phosphorylation of specific intracellular mediators (Hébert, 2011). The diversity of functions of FGF signaling are thought to stem from two broad, non-mutually exclusive hypotheses: the first suggests that different combinations of FGFs and FGFRs generate differential outcomes due to alternative signaling; the second proposes that different cell types respond to activated FGFRs in different ways, relative to one another (Hébert, 2011). Diverse FGF signaling is present in the central nervous system (CNS), as evidenced by a multitude of FGFs and three of the four FGFRs (FGFR1-FGFR3) (Bansal et al., 2003; Belluardo et al., 1997; El-Husseini, Paterson & Shiua, 1994). In embryonic mice lacking all three FGFRs in the CNS, there was a significant decrease in the volume and surface area of the dorsal telencephalon, attributable to a disruption of FGF signaling, thereby decreasing Notch expression in the early cortical ventricular zone (VZ) (Kang & Hebert, 2015). Without Notch expression, the proliferative stem-cell pool decreased because the stem cells prematurely exited the self-renewing mitotic cycle and thus, generated too many intermediate, differentiating cells (Rash et al., 2011). FGF signaling in the CNS is implicated in neural tube induction, the determination of neuronal and glial cell fate, adult neurogenesis, corpus callosum formation, cerebellar development, and maturation of interneurons. Aberrant FGF signaling is associated with neuropsychiatric disorders including major depressive disorder (MDD) and schizophrenia (Volk, Edelson & Lewis, 2016; Kang & Hebert, 2015; Smith et al., 2014; Elsayed et al., 2012; Smith et al., 2012; Hébert, 2011; Vaccarino et al., 2009; Gaughran et al., 2006; Smith et al., 2006; Alvarez, Araujo & Nieto, 1998; Rodriguez-Gallardo et al., 1997).

FGFR1 is one of the three FGFRs present in the CNS. Previous studies utilizing *in situ* hybridization have shown that *Fgfr1* is expressed in the embryonic hippocampal primordium, choroid plexus, cortical VZ, and cortical midline (Bansal et al., 2003; El-Husseini, Paterson & Shiua, 1994; Ohkubo et al., 2004; Smith et al., 2006). Furthermore, previous studies have shown that when FGF2, one of the primary ligands to FGFR1, is injected into the lateral ventricles of E15.5 rat embryos, there is a 53% increase in cortical volume and a 67% increase in total cell number at five days post-injection, as compared to vehicle injected controls (Vaccarino et al., 1999). Moreover, in *Fgf2* knockout mice, a marked decrease in the volume of the dorsal pseudostratified ventricular epithelium arises due to a reduction in the progenitor cell pool, which later results in a decreased abundance of cortical glutamatergic neurons in the frontal and parietal cortex (Korada et al., 2002; Raballo et al., 2000). In a transgenic mouse model with a *humanGFAP-Cre* (*hGFAP-Cre*) transgene driving targeted inactivation of a loxP-flanked *Fgfr1* allele (*Fgfr1*^*f*∕*f*;*hGFAPCre*^) there was an interruption of glial translocation from the dorsomedial VZ (glial wedge) to the prospective indusium griseum, an important source for axon guidance molecules. The indusium griseum failed to form, resulting in a disruption in the development of the hippocampal commissure and corpus callosum (Smith et al., 2006; Tole et al., 2006). Also in the *Fgfr1*^*f*∕*f*;*hGFAPCre*^ line, a significant reduction in hippocampal size and volume was observed, due to a decrease in dividing progenitor cells in the dentate gyrus (DG) and VZ of the hippocampus (Kang & Hebert, 2015; Ohkubo et al., 2004). Combined, these data indicate FGF/FGFR signaling is essential to the development of multiple brain structures, such as the cortex, hippocampus, corpus callosum, and indusium griseum. FGF signaling influences the abundance of proliferative cells, as well as the ability of glial cells to translocate (Ohkubo et al., 2004; Raballo et al., 2000; Smith et al., 2006; Vaccarino et al., 1999). Furthermore, FGFR1, in conjunction with FGFR2, has been shown to be critical in the development and morphology of the cerebellum, as evident in a hGFAP-Cre driven FGFR1/FGFR2 double knockout mouse model (hGFAP-Cre;Fgfr1^f∕f^;Fgfr2^f∕f^), by ensuring correct Bergmann glia morphology and abundance, which is essential for granule cell migration, and by influencing the proliferation of granule neuron precursors in the external granule layer (Smith et al., 2012). In the absence of appropriate FGFR1 signaling, there is a decrease in the abundance of interneurons by interfering with maturation of parvalbumin (PV) positive GABAergic interneurons. With too few PV positive interneurons, animals exhibit hyperactivity (Smith et al., 2008; Smith et al., 2014). Interestingly, both hyperactivity and decreased interneuron abundances co-occur in patients with schizophrenia, bipolar disorder, and Tourette’s syndrome (Volk & Lewis, 2013; Gonzalez-Burgos, Fish & Lewis, 2011; Kataoka et al., 2010; Hashimoto et al., 2008; Akbarian & Huang, 2006; Kalanithi et al., 2005; Benes et al., 2000; Volk et al., 2000). The *Fgfr1* gene has also been implicated in human conditions including Kallmann Syndrome (Anosmia and hypogonadotropic hypogonadism) and a craniostenosis syndrome, Pfeiffer syndrome (Villanueva & De Roux, 2010; Robin, Falk & Haldeman-Englert, 1998; Dodé et al., 2003). A better understanding of the biology of FGFR1 will follow the identification of cell types that express *Fgfr1* during neurodevelopment. This information could help guide the investigation of FGFR1 in neuropsychiatric disorders.

In previous ground-breaking studies performed by numerous investigators, most information about *Fgfr1* expression was obtained by utilizing *in situ* hybridization, which, unfortunately, lacks cell-type resolution, or by immunostaining with antibodies against FGFR1, for which specificity of the primary antibody can be problematic such that the antibodies cross react with other FGFRs (Bansal et al., 2003; Belluardo et al., 1997; Blak et al., 2005; Gonzalez et al., 1995; Ohkubo et al., 2004). Therefore, in order to accurately determine which cell types express *Fgfr1*, a transgenic reporter line was obtained from GENSAT. The *tgFGFR1-EGFPGP338Gsat* bacterial artificial chromosome (BAC) line (hereafter referred to as *tgFgfr1-EGFP*+) has the gene encoding enhanced green florescent protein (EGFP) driven by a transgenic *Fgfr1* promoter, and thus, under the same regulation and control as the endogenous *Fgfr1* promoter, it allows us to use green fluorescence protein (GFP) as an indicator of *Fgfr1* expression. Using the *tgFgfr1-EGFP+* transgenic line, we previously showed that PV+ interneurons did not colocalize with GFP+ cells, suggesting the loss of PV+ interneurons in mutants with inactivated *Fgfr1* occurs in a non-cell-autonomous manner (Smith et al., 2014). In the present study, we aimed to validate the sites of *Fgfr1* expression reported in previous studies that utilized *in situ* hybridization in embryonic mice, and then to extend those studies by determining which cell types in the developing mouse CNS express *Fgfr1.* These data were obtained by using immunocytochemistry to detect GFP using reliable anti-GFP antibodies. Furthermore, we aimed to establish the utility of the *tgFgfr1-EGFP+* line as a tool for future investigations of FGFR1 during embryonic development. To do this research, we utilized embryonic day 14.5 (E14.5) and postnatal day 0.5 (P0.5) mice from the *tgFgfr1-EGFP+* line, along with immunostaining and fluorescence microscopy.

Presently, we show that in E14.5 *tgFgfr1-EGFP+* mice there is co-localization of GFP and brain lipid-binding protein (BLBP) in the radial glial cells of the VZ, cortical midline, lateral ganglionic eminence (LGE), lateral pallial-subpallial boundary, and cerebellar anlage. Furthermore, we also find co-localization of GFP and doublecortin (DCX) in neuroblasts of the developing cortex and hippocampal primordium of E14.5 *tgFgfr1-EGFP+* mice. We also investigated cell type expression of *Fgfr1* in P0.5 *tgFgfr1-EGFP+* mice. Our studies indicate co-localization of GFP with BLBP+ radial glia in the VZ of the cortex, hippocampus, hypothalamus, and glial wedge, and also throughout the cerebellum. Co-localization with GFP can also be seen in astrocytes positive for glial fibrillary acidic protein (GFAP) within the corpus callosum, glial wedge, and indusium griseum, and in NeuN+ neurons within the anterior cingulate cortex.

## Materials and Methods

### Animals

The animals used in this study, *tgFGFR1-EGFPGP338Gsat* bacterial artificial chromosome (BAC) line, were created by the GENSAT project by microinjecting bacterial artificial chromosome with a Fgfr1 promoter driving EGFP into the pronucleus of fertilized mouse eggs, and obtained from the Mutant Mouse Resource Center (MMRRC.org) at UC Davis. This construct allowed us to map and identify the cells expressing *Fgfr1* in the developing CNS. The GENSAT project has the aim of creating a library of transgenic BAC lines in order to map the most important genes in the CNS (Heintz, 2004). Control mice (*tgFgfr1-EGFP-*) lacked the EGFP transgene and were thus EGFP negative. All animals used in this study were treated humanely and ethically in accordance to the recommendations from The Guide for the Care and Use of Laboratory Animals of the National Institutes of Health. All euthanasia was conducted ethically and humanely to minimize suffering, as outlined under the University of Louisiana at Lafayette IACUC committee APS number 2013- 8717-053.

### Genotyping

In order to determine if the mice contained the *tgFGR1-EGFPGP338Gsat* gene construct, we conducted polymerase chain reactions (PCR) for EGFP or by screening with goggles that contained a GFP filter (BLS LTD). The following procedure for PCR based genotyping was used: tails of mice were collected and DNA was extracted from the tail using 50mM sodium hydroxide (95 °C for 30 min), followed by neutralization with 1M TRIS (pH 7.6). Master mix for 1 reaction of PCR for amplifying *EGFP* was created using 2.5 µl of 10× PCR buffer, 0.5 µl of 10 mM dNTP mix, 1 µl of forward and reverse primer mix (Forward: AAGTTCATCTGCACCACCG and Reverse: TGCTCAGGTAGTGGTTGTCG ), 0.2 µl of 5 units/µl Hot start Taq Polymerase and 18.8 µl of distilled water. 2 µl of DNA sample were added to 23 µl of Master mix per PCR tube and the samples were amplified in the Applied Biosystems 96 Well Thermocycler.

### Immunostaining

Embryos were obtained from timed pregnancies based on identification of a vaginal mucus plug (Embryonic Date = 0.5 on morning found). Pregnant dams were euthanized by CO_2_ inhalation at E14.5, the uterine horn was then removed and placed in cold Hank’s balanced salts solution (HBSS). Embryos were dissected in buffer and placed in 4% paraformaldehyde (PFA) in 1× phosphate buffered saline (PBS) overnight at 4 °C, followed by cryoprotection in 20% sucrose/1X PBS at 4 °C overnight. P0.5 mice were anesthetized by being placed on a metal plate on ice for 10–15 min to induce hypothermia followed by decapitation. P0.5 brains were dissected and fixed as described for embryos. E14.5 and P0.5 mouse brains were cryopreserved in OCT within cryomolds and placed in a dry ice/ethanol bath. We then stored all samples at −80 °C. Embryonic and P0.5 brains were thin sectioned coronally (20 µm onto superfrost slides) in a cryostat (Microm, HM 505 E) and stored at −80 °C until immunostaining.

Slides were removed from −80 °C and allowed to equilibrate to room temperature. We then removed any excess OCT from the slides. Using an ImmEdge™ pen (Vector Laboratories, Inc., Burlingame, CA, USA) we circumscribed each tissue section and allowed it to dry. We then blocked the tissue by adding a droplet of 10% normal goat serum (NGS) in 1xPBS with 0.01% Tween (Sigma Aldrich, St. Louis, MO, USA) and 0.02% TritonX (Sigma Aldrich, St. Louis, MO, USA). The appropriate primary antibodies in 5% NGS in 1xPBS with 0.01% Tween (Sigma Aldrich, St. Louis, MO, USA) and 0.02% TritonX (Sigma Aldrich, St. Louis, MO, USA) (Table 1) were added and allowed to incubate overnight at 4 °C. The sections were washed, and primary antibodies detected with Alexa conjugated secondary antibodies (Jackson Labs and Abcam, Cambridge, UK) in 5% NGS in 1xPBS with 0.01% Tween and 0.02% TritonX and allowed to incubate for 2 h, followed by a wash with 1xPBS. Coverslips were placed on the slides with VECTASHIELD DAPI as the mounting medium.

**Table 1:** Primary antibodies used in immunostaining.

Antigen	Raised in	Dilution	Source	Marker of	Catalogue #
BLBP	Rabbit	1:500	Abcam Inc.	Glial stem cells	AB32423
DCX	Mouse	1:500	Abcam Inc.	Neuroblasts	AB18723
GFAP	Rabbit	1:500	Dako Cytomation	Astrocytes	Z0334
GFP	Chicken	1:500	Abcam Inc.	GFP	AB13970
NeuN	Mouse	1:250	Millipore	Neurons	MAB377
Tbr2	Rabbit	1:1,000	Abcam Inc.	Intermediate precursors	AB23345

### Tissue clearing

After sacrificing the pups to obtain brain samples, the paws were processed for tissue clearing using the CLARITY technique (Chung et al., 2013).

### Fluorescence microscopy

Fluorescent secondary antibodies in immunostained tissue were imaged using Stereo Investigator software (MBF Biosciences, Williford, VT, USA) coupled with a AxioCam MRm on the Zeiss Axioimager M2 microscope equipped with an ApoTome.2. Tissue cleared using the CLARITY technique was imaged as described above. In order to determine the presence of double labeled cells in immunostained tissue, single and *z*-stack images were obtained and observed as separate channels as well as in composite images. Fluorescence microscopy using a Nikon SMZ18 stereo microscope was used to image paws at low magnification.

## Results

### *Fgfr1* is expressed in the developing E14.5 and perinatal P0.5 mouse brain

**Figure 1:**
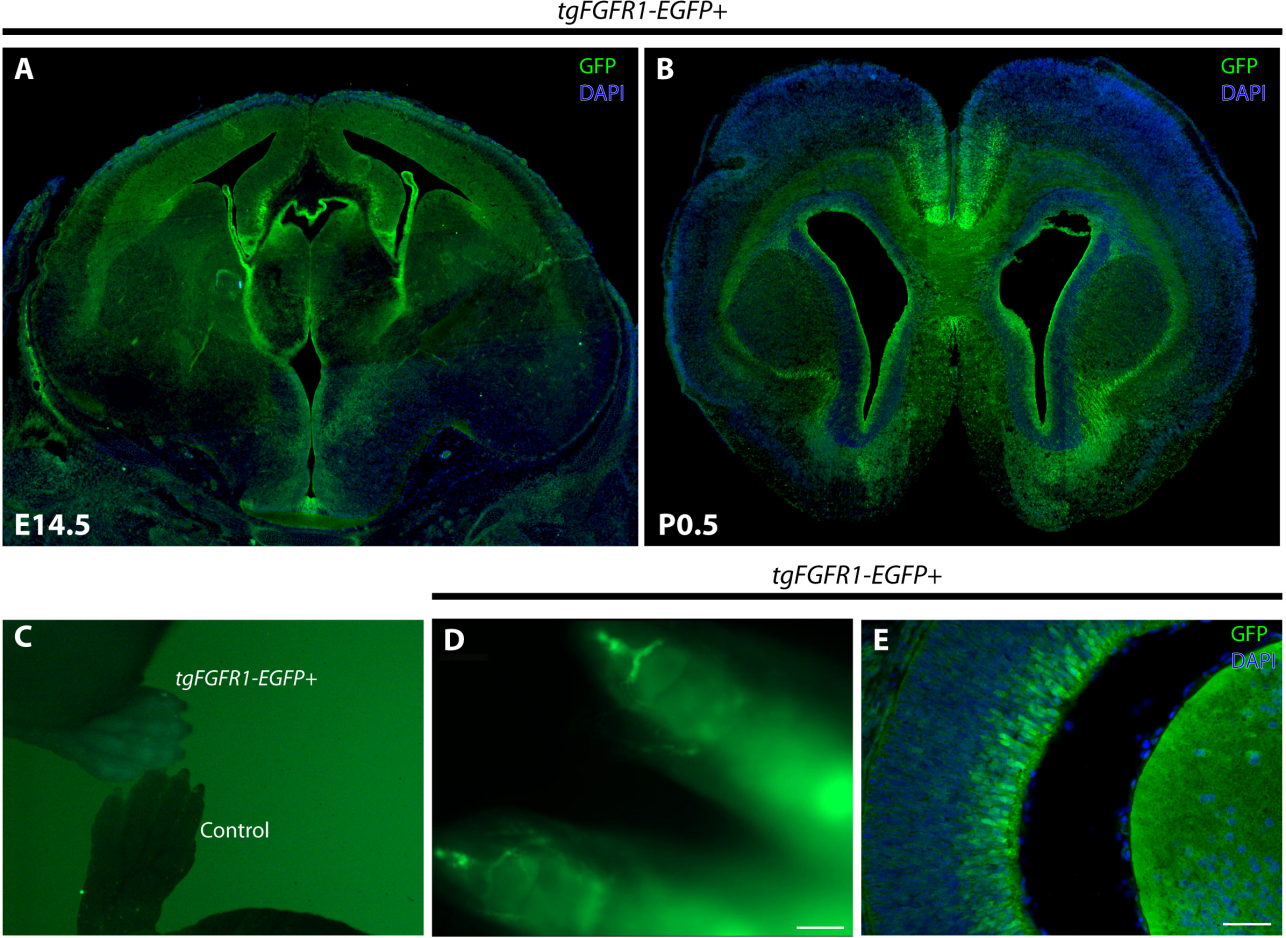
*Fgfr1* is expressed in various regions throughout the developing E14.5 and perinatal P0.5 mouse brain. GFP immunostaining with DAPI counterstaining of *tgFgfr1-EGFP+* mice coronal sections (A, B, and E) indicate *Fgfr1* expression in the VZ and upper layers of the developing dorsal and cingulate cortex, the VZ and intermediate zone of the hippocampal primordium, the choroid plexus, and the hypothalamus at E14.5 (A); and in the VZ surrounding the lateral ventricles, anterior cingulate cortex, indusium griseum, glial wedge, and in the lateral pallial-subpallial boundary at P0.5 (B). Low-power fluorescence imaging revealed GFP fluorescence in the paws of *tgFgfr1-EGFP+* mice as compared to a control (C). Tissue clearing via the CLARITY technique revealed GFP fluorescence in the growth plates of the distal paw (D). GFP expression was also observed in the developing eye at E14.5 (E). Scale bars = 200 µm in (D) and 50 µm in (E).

*Fgfr1* expression at E14.5, evident by a positive GFP signal, can be seen in the VZ and upper layers of the developing dorsal and cingulate cortex, the VZ and intermediate zone of the hippocampal primordium, the choroid plexus, and the hypothalamus (Fig. 1A). By using the *tgFgfr1-EGFP+* mouse line, *Fgfr1* expression at P0.5, as apparent by a positive GFP signal in immunostained *tgFgfr1-EGFP+* mice, can be seen in the VZ surrounding the lateral ventricles, anterior cingulate cortex, indusium griseum, glial wedge, and in the lateral pallial-subpallial boundary (Fig. 1B). *Fgfr1* expression can also be observed in regions other than the brain, such as the postnatal growth plates of the distal limbs (Figs. 1C and 1D) and in the apparent Müller glia of the eyes as well as in the lens (Fig. 1E). Since we expected to see GFP in the growth plates, and a GFP signal was observed in all four paws when we imaged the pups under the fluorescence dissection microscope (Fig. 1C), we performed optical clearing of the dissected limbs according to the protocol by Chen et al., 2013 and imaged the cleared tissue to observe expression (Fig. 1D).

In order to elucidate the cellular identity of the *Fgfr1* expressing GFP+ cells in the developing nervous system of E14.5 mice embryos, coronal sections of *tgFgfr1-EGFP+* and control mice brains were immunostained for BLBP+ radial glial cells, Tbr2+ intermediate progenitor cells (IPCs), and DCX+ neuroblasts, each in conjunction with GFP immunostaining and DAPI counterstaining. To further proceed with our investigation of *Fgfr1* expression, we also studied the perinatal nervous system of P0.5 mice to determine the cellular identity of the *Fgfr1* expressing GFP+ cells by immunostaining coronal sections of *tgFgfr1-EGFP+* and control mice brains for BLBP+ radial glial cells, GFAP+ astrocytes, and NeuN+ neurons, each in conjunction with GFP immunostaining and DAPI counterstaining. In all further figures, cell-specific markers were visualized with red fluorescence, the *Fgfr1* expressing GFP+ cells were visualized with green fluorescence , and the DAPI counterstain was visualized with blue fluorescence , while co-localized cells will appear to fluoresce yellow or orange due to the dual red (cell-specific marker) and green (*Fgfr1* expressing cell) fluorescence given off by double stained cells.

### *Fgfr1* is expressed in BLBP+ cells of various regions, and DCX+ cells of the cortex and hippocampal primordium at E14.5

**Figure 2:**
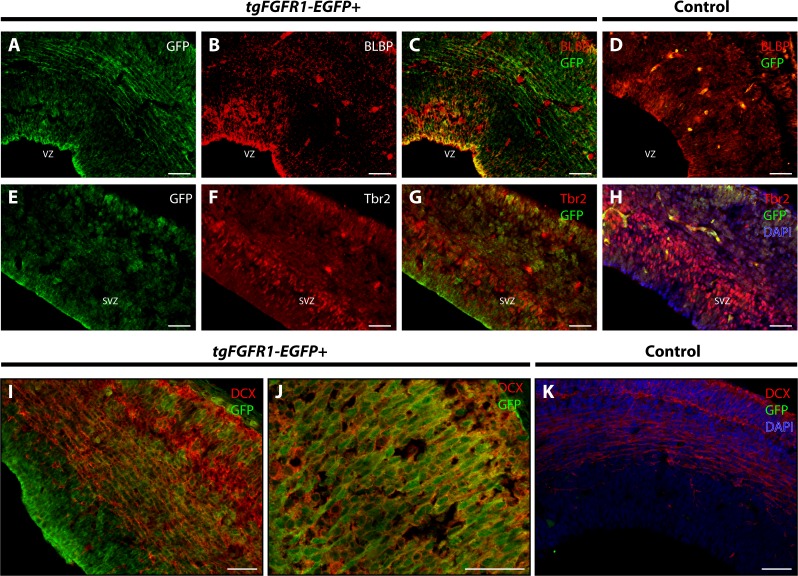
*Fgfr1* is expressed in BLBP+ and DCX+ cells of the developing cortex at E14.5. Immunostaining for BLBP and GFP in E14.5 *tgFgfr1-EGFP+* (A–C) and control (D) mice revealed co-localization within the VZ of the dorsal cortex (A–C, with (A) and (B) showing separated green (GFP) and red (BLBP) channels, respectively). Immunostaining for Tbr2 and GFP in E14.5 *tgFgfr1-EGFP+* (E–G) and control (H) mice revealed no observable co-localization within the dorsal cortex (E–G, with (E) and (F) showing separated green (GFP) and red (Tbr2) channels, respectively). Immunostaining for DCX and GFP in E14.5 *tgFgfr1-EGFP+* (I and J) and control (K) mice revealed co-localization within the dorsal cortex in figures (I) (low magnification) and (J) (high magnification). Controls H and K also feature DAPI counterstaining, with DAPI staining omitted from D (D, H, and K). All scale bars are 50 µm. VZ, ventricular zone; SVZ, subventricular zone.

BLBP and GFP immunostaining showed strong co-localization of BLBP+ radial glial cells with GFP in the dorsal VZ of the developing cortex of *tgFgfr1-EGFP+* mice (Figs. 2A–2C, and Supplemental Information 1). Immunostaining for Tbr2+ intermediate progenitor cells and *Fgfr1* expressing GFP+ cells revealed no observable co-localization of Tbr2 and GFP within the dorsal cortex of E14.5 *tgFgfr1-EGFP+* mice (Figs. 2E–2G). In the dorsal cortex of *tgFgfr1-EGFP+* mice, a large number of GFP+ cells were also immunostained with DCX+ neuroblasts (Figs. 2I and 2J). All control (*tgFgfr1-EGFP-*) mice presented little to no GFP+ signal in immunostained preparations of brain tissue (Figs. 2D, 2H and 2K).

**Figure 3:**
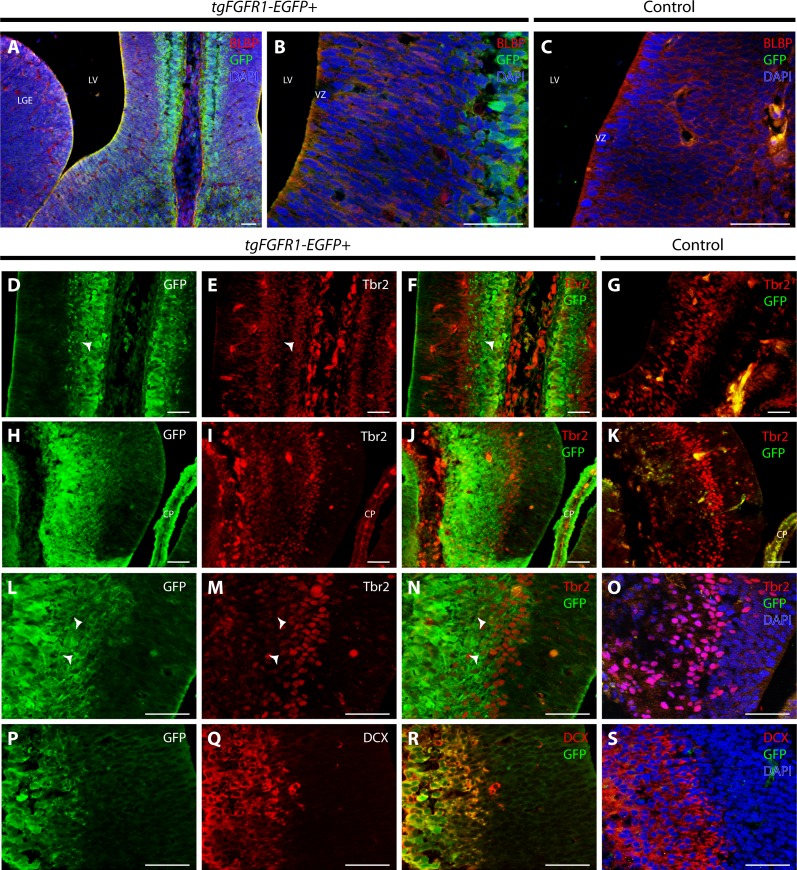
*Fgfr1* is expressed in midline structures at E14.5. Immunostaining for BLBP and GFP with DAPI counterstaining in E14.5 *tgFgfr1-EGFP+* (A and B) and control (C) mice revealed co-localization within the cells of the midline VZ in figures A (low magnification) and B (high magnification). Immunostaining for Tbr2 and GFP in midline structures of E14.5 *tgFgfr1-EGFP+* (D–F, H–J, and L–N) and control (G, K, and O) mice revealed minimal co-localization within the cingulate cortex ((D–F), with (D) and (E) showing separated green (GFP) and red (Tbr2) channels, respectively) and hippocampal primordium ((H–J) (low magnification) and (L–N) (high magnification), with (H) and (L) showing separated green (GFP), and (I) and (M) showing separated red (Tbr2) channels). Immunostaining for DCX and GFP in E14.5 *tgFgfr1-EGFP+* (P–R) and control (S) mice, which also features DAPI counterstaining, revealed co-localization within the intermediate zone of the hippocampal primordium ((P–R), with (P) and (Q) showing separated green (GFP) and red (DCX) channels, respectively). All scale bars = 50 µm. LGE, lateral ganglionic eminence; LV, lateral ventricle; VZ, ventricular zone; CP, choroid plexus; Arrowheads indicate double labeled cells.

There was also co-localization of BLBP+ radial glial cells with GFP in the VZ of the cingulate cortex at E14.5 in *tgFgfr1-EGFP+* mice (Figs. 3A and 3B). Contrary to dorsal cortex, immunostaining for Tbr2+IPCs and *Fgfr1* expressing GFP+ cells revealed an observable, albeit dim co-localization of Tbr2 and GFP in the cingulate cortex of *tgFgfr1-EGFP+* mice (Figs. 3D–3F) as well as in the intermediate zone of the hippocampal primordium of *tgFgfr1-EGFP+* mice (Figs. 3H–3J, low magnification and 3L–3N, high magnification). However, the majority of *Fgfr1* expressing GFP+cells in the intermediate zone of the hippocampal primordium at E14.5 were highly co-localized with neuroblasts positive for DCX in *tgFgfr1-EGFP+* mice (Figs. 3P–3R). All control mice (*tgFgfr1-EGFP-)* presented little to no GFP+ signal in the immunostainings that were conducted (Figs. 3C, 3G, 3K, 3O and 3S).

**Figure 4:**
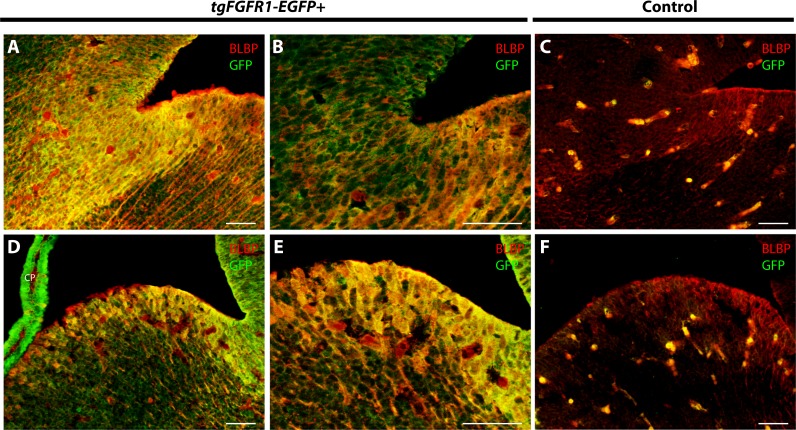
*Fgfr1* is expressed in the LGE and lateral pallial-subpallial boundary at E14.5. Immunostaining for BLBP and GFP in E14.5 *tgFgfr1-EGFP+* (A, B, D, and E) and control (C and F) mice, revealed co-localization in the lateral pallial-subpallial boundary, shown in figures (A) (low magnification) and (B) (high magnification), and in the LGE, shown in figures (D) (low magnification) and (E) (high magnification). All scale bars = 50 µm. CP, choroid plexus.

To further proceed with our investigation of *Fgfr1* expression in BLBP+ radial glial cells, we examined both the lateral pallial-subpallial boundary and LGE of the developing E14.5 mouse brain. Immunostaining for BLBP+ radial glial cells and *Fgfr1* expressing GFP+ cells revealed high apparent co-localization of GFP and BLBP within the cell bodies and projections in the lateral pallial-subpallial boundary of *tgFgfr1-EGFP+* mice (Figs. 4A and 4B). Furthermore, strong levels of co-localization could be observed within the cell bodies and projections of BLBP+/GFP+ cells in the LGE of *tgFgfr1-EGFP+* mice immunostained for BLBP and GFP (Figs. 4D and 4E). All control mice (*tgFgfr1-EGFP-)* presented little to no GFP+ signal in the immunostained sections (Figs. 4C and 4F).

### *Fgfr1* is expressed in BLBP+ stem cells of the cerebellar anlage at E14.5

**Figure 5:**
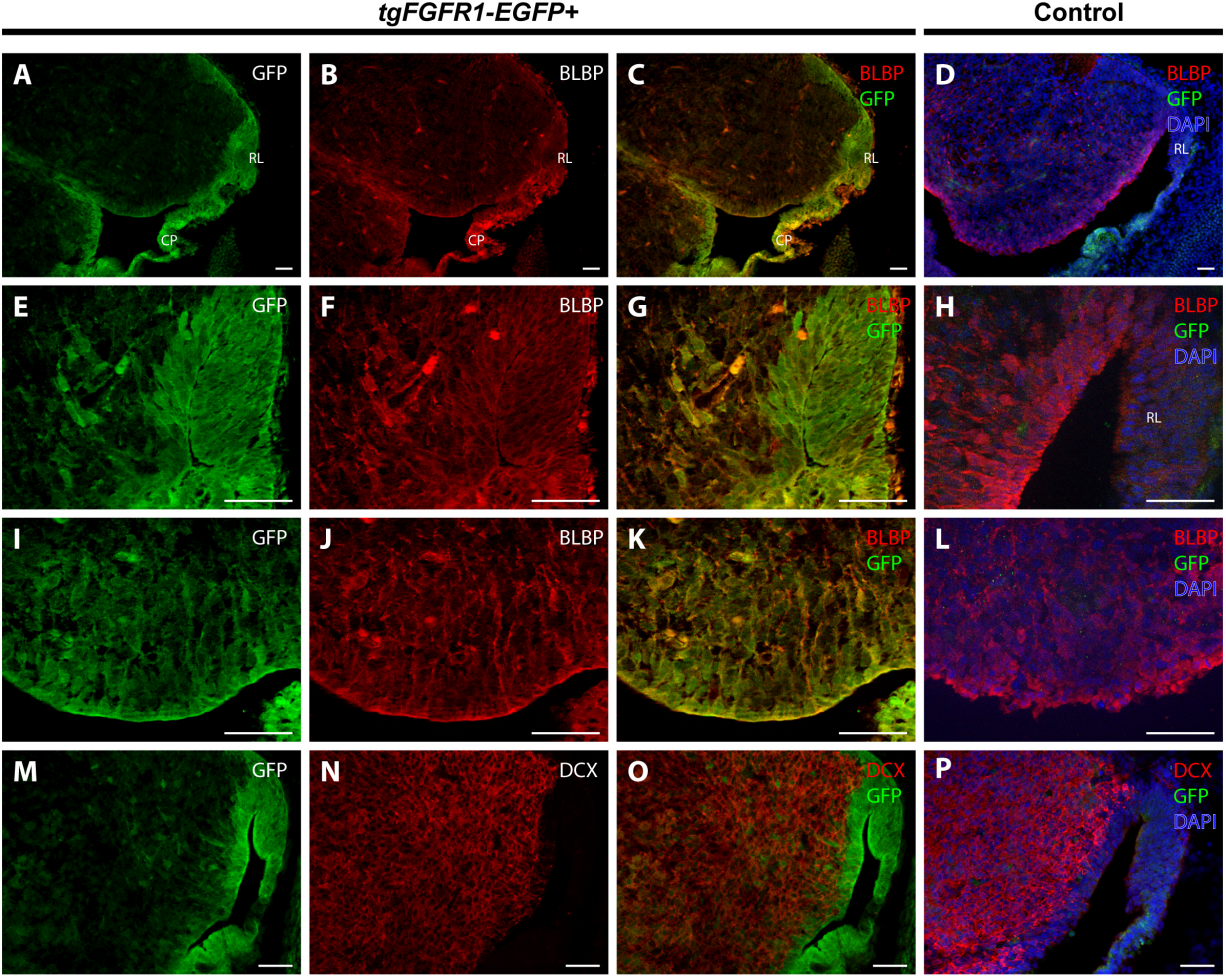
*Fgfr1* is expressed in the cerebellar anlage at E14.5. Immunostaining for BLBP and GFP in E14.5 *tgFgfr1-EGFP+* (A–C, E–G, and I–K) and control (D, H, and L) mice, which also feature DAPI counterstaining, revealed co-localization in the rhombic lip, shown in (A–C) (low magnification, with (A) and (B) showing separated green (GFP) and red (BLBP) channels, respectively) and (E–G) (high magnification, with (E) and (F) showing separated green (GFP) and red (BLBP) channels, respectively); the cerebellar neuroepithelium, shown in (A–C) (low magnification, with (A) and (B) showing separated green (GFP) and red (BLBP) channels, respectively) and (I–K) (high magnification, with (I) and (J) showing separated green (GFP) and red (BLBP) channels, respectively). Immunostaining for DCX and GFP in E14.5 *tgFgfr1-EGFP+* (M–O) and control (P) mice, which also feature DAPI counterstaining, revealed no co-localization within the rhombic lip or cerebellar neuroepithelium, and very little within the interior of the cerebellar anlage ((M–O), with (M) and (N) showing separated green (GFP) and red (DCX) channels, respectively) All scale bars = 50 µm. RL, rhombic lip. CP, choroid plexus.

In order to determine the cell types expressing *Fgfr1* in the cerebellar anlage, we immunostained *tgFgfr1-EGFP+* mice, in addition to control mice, for BLBP+ radial glial cells, DCX+ neuroblasts, and *Fgfr1* expressing GFP+ cells. Immunostaining of *tgFgfr1-EGFP+* mice for BLBP+ stem cells and *Fgfr1* expressing GFP+ cells revealed co-localization of BLBP and GFP in cell bodies of the rhombic lip (Figs. 5A–5C and 5E–5G) and the ventricular zone of the cerebellar anlage, which showed abundant co-localization in the cell bodies and projections (Figs. 5A–5C and 5I–5K). We also immunostained for DCX+ neuroblasts and *Fgfr1* expressing GFP+ cells. We observed DCX+ neuroblasts within interior regions of the cerebellar anlage with a largely non-overlapping pattern of *Fgfr1* expressing GFP+ cells within the rhombic lip and ventricular zone (Figs. 5M–5O). All control mice (*tgFgfr1-EGFP-)* presented little to no GFP+ signal (Figs. 5D, 5H, 5L and 5P).

### *Fgfr1* is expressed in various cell types in the anterior dorsal midline at P0.5

**Figure 6:**
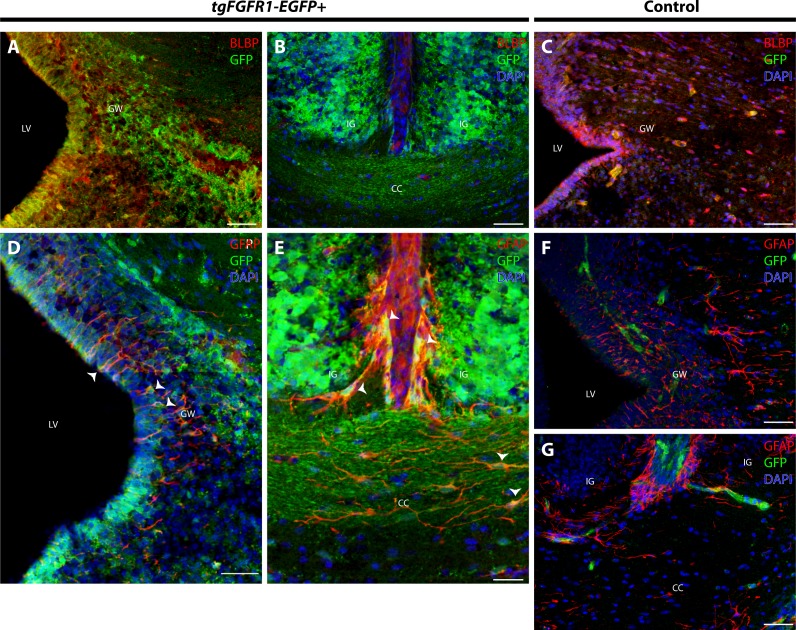
*Fgfr1* is expressed in anterior midline structures at P0.5. Immunostaining for BLBP and GFP in P0.5 *tgFgfr1-EGFP+* (A and B), with DAPI counterstaining in (B), and control (C) mice, which also features DAPI counterstaining, revealed co-localization in the VZ of the glial wedge (A), but not in the indusium griseum (B) in the doral midline of the developing cortex. Immunostaining for GFAP and GFP with DAPI counterstaining in P0.5 *tgFgfr1-EGFP+* (D and E) and control (F and G) mice revealed co-localization in the glial wedge (D), indusium griseum (E), and corpus callosum (E). All scale bars = 50 µm. LV, lateral ventricle; GW, glial wedge; CC, corpus callosum; IG, indusium griseum; Arrowheads indicate double labeled cells.

In order to elucidate which cell types of the anterior dorsal midline express *Fgfr1,* we immunostained *tgFgfr1-EGFP+* and control mice for BLBP+ radial glial cells, GFAP+ astrocytes, and NeuN+ neurons in the indusium griseum and glial wedge. Immunostaining for BLBP+ radial glia and *Fgfr1* expressing GFP+ cells revealed strong co-localization of BLBP and GFP in the glial wedge (Fig. 6A), but not in the indusium griseum (Fig. 6B). Immunostaining for GFAP+ astrocytes and *Fgfr1* expressing GFP+ cells revealed co-localization of GFAP+ astrocytes with GFP in the glial wedge of *tgFgfr1-EGFP+* mice (Fig. 6D, Fig. S2). There was also co-localization of GFAP+ astrocytes with GFP in the corpus callosum and indusium griseum (Fig. 6E) of P0.5 *tgFgfr1-EGFP+* mice immunostained for GFAP and GFP. All control mice (*tgFgfr1-EGFP-)* presented little to no GFP+ signal (Figs. 6C, 6F and 6G).

**Figure 7:**
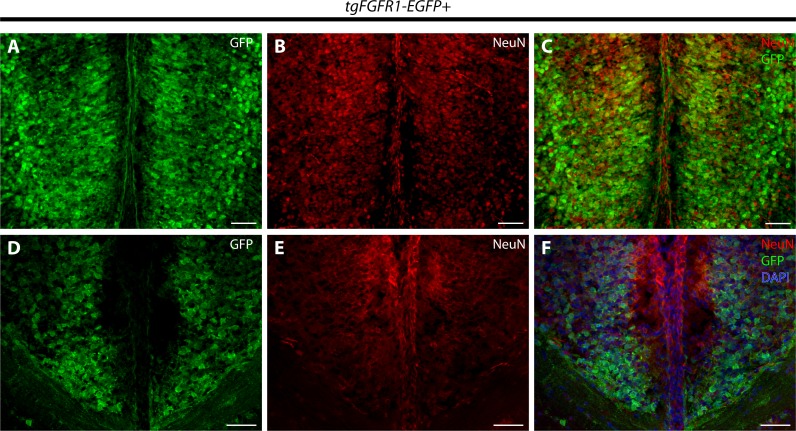
*Fgfr1* is expressed in NeuN+ neurons of the cingulate cortex at P0.5. Immunostaining for NeuN and GFP in P0.5 *tgFgfr1-EGFP+* mice (A–F), with DAPI counterstaining in (F), revealed some co-localization of NeuN and GFP in the cingulate cortex ((A–C), with (A) and (B) showing separated green (GFP) and red (NeuN) channels, respectively), but none in the indusium griseum ((D–F), with (D) and (E) showing separated green (GFP) and red (NeuN) channels, respectively). All scale bars = 50 µm.

Immunostaining for NeuN+ neurons and *Fgfr1* expressing GFP+ cells of P0.5 *tgFgfr1-EGFP+* mice, revealed co-localization of NeuN and GFP within cells of the anterior cingulate cortex (Figs. 7A–7C). In contrast to what was observed with GFAP staining, when NeuN and GFP immunostaining was examined in the indusium griseum of P0.5 *tgFgfr1-EGFP+* mice, we found little to no indication of NeuN+ neurons within the indusium; however, *Fgfr1* expressing GFP+ cells were found to be present (Figs. 7D–7F).

### *Fgfr1* expression in the P0.5 perinatal hippocampus and cortex

**Figure 8:**
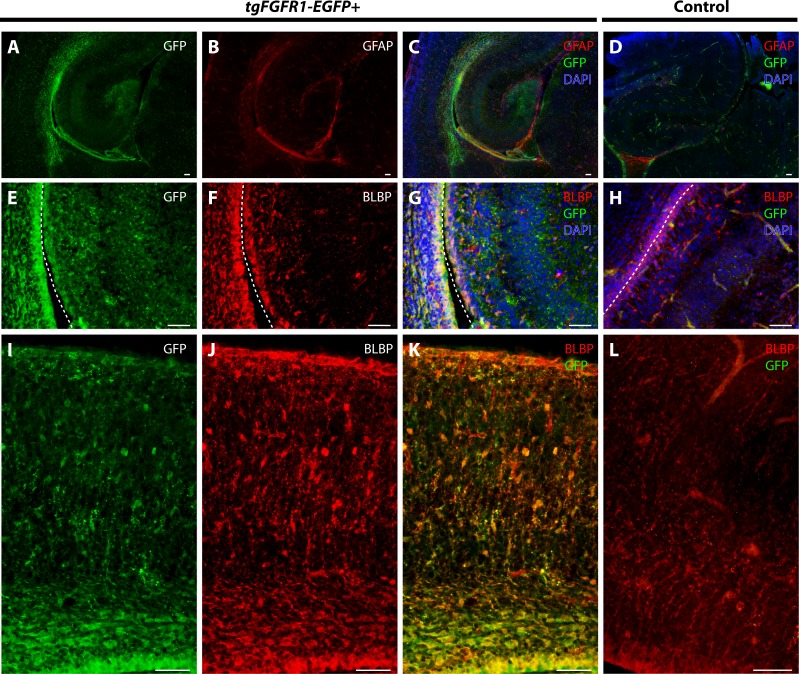
*Fgfr1* is expressed in BLBP+ cells in the hippocampus and cortex at P0.5. Immunostaining for GFAP and GFP with DAPI counterstaining in P0.5 *tgFgfr1-EGFP+* (A–C) and control (D) mice revealed no observable co-localization within the hippocampus at this age ((A–C), with (A) and (B) showing separated green (GFP) and red (GFAP) channels, respectively). Immunostaining for BLBP and GFP in P0.5 *tgFgfr1-EGFP+* (E–G and I–K) and control mice (H and L), with DAPI counterstaining in (G) and (H), revealed co-localization in the hippocampal VZ and CA ((E–G), with (E) and (F) showing separated green (GFP) and red (GFAP) channels, respectively), and throughout the cortex ((I–K), with (I) and (J) showing separated green (GFP) and red (GFAP) channels, respectively from images obtained in the dorsal cortex). In figures (E–H) the hippocampal VZ is on the right side of the dotted line, which separates the hippocampal and cortical VZ. All scale bars = 50 µm.

We examined *Fgfr1* expression in the P0.5 hippocampi of *tgFgfr1-EGFP+* mice immunostained for GFAP+ astrocytes and BLBP+ radial glia. At P0.5, GFAP positive immunostaining has not attained the same level as adult brains, and most GFAP signal is observed in the midline glial structures (Figs. 6D–6F). GFAP and GFP immunostaining revealed that GFAP+astrocytes were not found in the hippocampus; notably, however, there were *Fgfr1* expressing GFP+ cells in the hippocampus and cortex (Figs. 8A–8C). Immunostaining for BLBP and GFP in the hippocampus showed co-localization of BLBP+ radial glia with GFP in the VZ and cornu ammonis (CA) region of P0.5 *tgFgfr1-EGFP+* mice (Figs. 8E–8G); however, there was no observable BLBP signal detected in the dentate gyrus at this age (DG not shown in figure). BLBP and GFP immunostaining of P0.5 *tgFgfr1-EGFP+* mice revealed a high occurrence of co-localization of GFP within BLBP+ radial glia throughout the layers of the cortex (Figs. 8I–8K). All control mice (*tgFgfr1-EGFP-)* presented little to no GFP+ signal (Figs. 8D, 8H and 8L).

### *Fgfr1* is expressed in BLBP+ radial glia of the hypothalamus, cerebellum, and lateral pallial-subpallial boundary at P0.5

**Figure 9:**
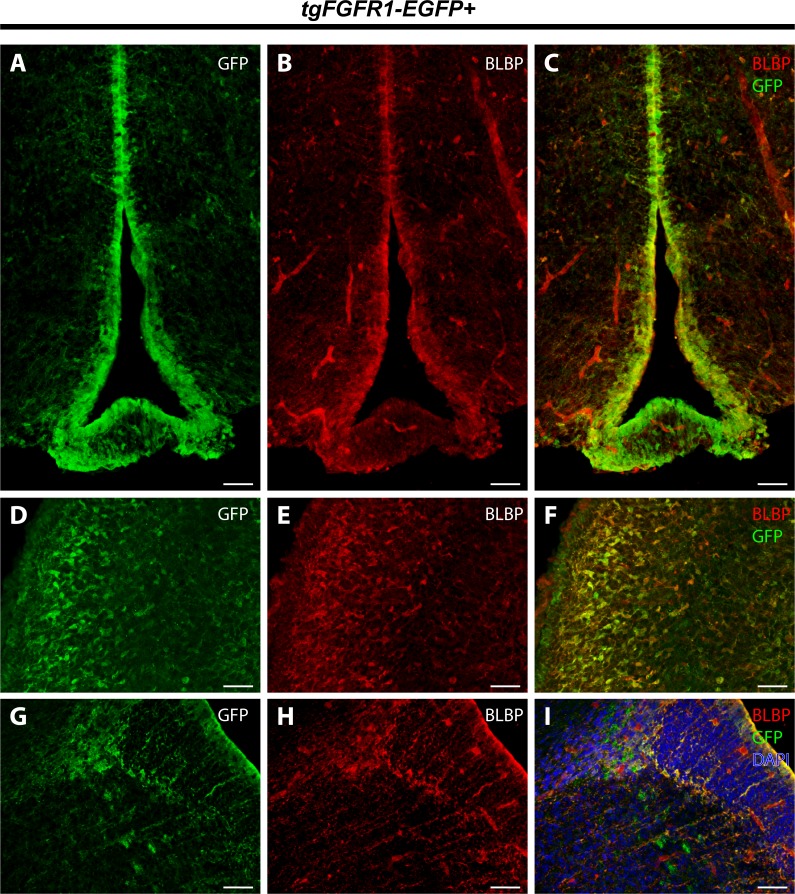
*Fgfr1* is expressed in BLBP+ cells of the hypothalamus, cerebellum, and lateral pallial-subpallial boundary at P0.5. Immunostaining for BLBP and GFP in P0.5 *tgFgfr1-EGFP+* mice (A–I), with DAPI counterstaining in (I), revealed co-localization in the ventricular walls of the hypothalamus ((A–C), with (A) and (B) showing separated green (GFP) and red (BLBP) channels, respectively), within the cerebellum ((D–F), with (D) and (E) showing separated green (GFP) and red (BLBP) channels, respectively), and in the lateral pallial-subpallial boundary ((G–I), with (G) and (H) showing separated green (GFP) and red (BLBP) channels, respectively). All scale bars = 50 µm.

To further proceed with our investigation of *Fgfr1* expression in BLBP+ cells of the perinatal mouse brain, we examined BLBP+ glial cells and *Fgfr1* expressing GFP+ cells in the hypothalamus, cerebellum, and lateral pallial-subpallial boundary of P0.5 *tgFgfr1-EGFP+* mice. Here we show that BLBP+ radial glia along the hypothalamic VZ co-localized with GFP, as evident by the immunostaining for BLBP and GFP in P0.5 *tgFgfr1-EGFP+* mice (Figs. 9A–9C). Furthermore, we also show co-localization of BLBP and GFPwithin the perinatal cerebellum (Figs. 9D–9F) and in both the cell bodies and processes of BLBP+/GFP+ cells within and at the VZ of the lateral pallial-subpallial boundary (Figs. 9G–9H) of *tgFgfr1-EGFP+* mice.

## Discussion

The *tgFgfr1-EGFP+* line provides simple, yet accurate cell-type identification of *Fgfr1* expressing cells of the developing embryonic and perinatal brain of both E14.5 and P0.5 mice via immunostaining for cell-specific markers in combination with immunostaining for *Fgfr1* expressing GFP+ cells. In E14.5 brains, *Fgfr1* was highly expressed in BLBP+ radial glial cells of the cortical VZ, lateral pallial-subpallial boundary, LGE, rhombic lip, VZ of the cerebellar anlage. Furthermore, *Fgfr1* was highly expressed in DCX+ neuroblasts of the dorsal cortex and hippocampal primordium, but sparsely expressed in DCX+ neuroblasts of the cerebellar anlage. *Fgfr1* was also sparsely expressed in Tbr2+IPCs of the cingulate cortex and hippocampal primordium. In the brains of P0.5 mice, *Fgfr1* was highly expressed in BLBP+ cells of the cortex, glial wedge, hippocampus, hypothalamus, cerebellum, and lateral pallial-subpallial boundary, and in GFAP+ astrocytes of the glial wedge and indusium griseum. Additionally, *Fgfr1* was also expressed in the NeuN+ neurons of the cingulate cortex. Taken together, our data reveal *Fgfr1* is primarily expressed in proliferative glial stem cells throughout the embryonic and perinatal brain, in addition to astroglia likely undergoing soma translocation.

During CNS development, correct size and organization of the cortex and hippocampus results from a stringent, yet temporally dynamic balance between the self-renewal of radial glial stem cells and the production of their differentiated daughter cells. In an attempt to better understand the cellular mechanisms controlling the brain size and the numbers of neuronal and glial cells, investigations of FGFs, FGFRs, and their signaling have strongly suggested the importance of proper FGF signaling in maintaining the self-proliferative state of radial glial stem cells (Rash et al., 2011; Stevens et al., 2010; Kang et al., 2009; Thomson et al., 2009; Ohkubo et al., 2004). However, previous studies have been unable to accurately determine which cell types express FGFRs. Presently, we show strong expression of *Fgfr1* in BLBP+ radial glial stem cells throughout the VZ of the developing telencephalon at both E14.5 and P0.5 and in the hippocampal primordium and the more-developed hippocampus at E14.5 and P0.5, respectively. Furthermore, our findings indicate *Fgfr1* expression in DCX+ neuroblasts of the dorsal cortex and hippocampal primordium. These findings are consistent with the possibility that FGFR1 has a direct, cell-autonomous role in mediating the extracellular signals controlling the balance between the proliferative, self-renewing BLBP+ radial glial stem cells and their differentiating progeny in the telencephalon during embryonic and perinatal development.

We observed strong expression of *Fgfr1* in developing anterior midline within the developing cingulate cortex. FGF8 and FGF17 gradients that originate from the forebrain rostral patterning center are implicated in patterning of the frontal and cingulate cortex (Cholfin & Rubenstein, 2007; Cholfin & Rubenstein, 2008). While evidence suggests that FGF8 does not have a high binding affinity for FGFR1, it is interesting to note that FGF17 mutants and FGFR1/FGFR2 double mutants have reduced prefrontal cortex volume (Stevens et al., 2010; Cholfin & Rubenstein, 2007). Thus, FGF17 might be the more important signal for forebrain pattering. Abundant *Fgfr1* expression in the anterior cingulate has implications for psychiatric conditions. Elevated *Fgfr1* expression in the prefrontal cortex co-occurs with schizophrenia (Volk, Edelson & Lewis, 2016). Furthermore, FGF2 and FGFR1 signaling in the prefrontal cortex is implicated in human depression and in rodent models of depression (Elsayed et al., 2012; Bachis et al., 2008; Kang et al., 2007; Gaughran et al., 2006; Evans et al., 2004). The high expression of *Fgfr1* in the prefrontal cortex in neonatal brains may have significant implications towards understanding plasticity of the cortex and the effects of perinatal stress or insults upon future psychiatric risk.

Other areas abundant with proliferative and migrating cells are the pallial-subpallial boundary and LGE of the embryonic brain, which share the common functions of giving rise to interneurons destined for the cortex or olfactory bulbs by way of the rostral migratory stream, and providing axonal guidance by which these migrations are organized (Subramanian et al., 2009; Stenman, Toresson & Campbell, 2003). Similar to other areas plentiful in proliferation and migration of stem cells and their progeny, *Fgfr1* is expressed in BLBP+ cells of both the LGE and lateral pallial-subpallial boundary of E14.5 mice. Furthermore, *Fgfr1* expression was also seen in BLBP+ cells of the lateral pallial-subpallial boundary of P0.5 mice. These findings are consistent with the notion that *Fgfr1* is expressed in glial and stem cells of the embryonic and perinatal nervous system.

Two factors essential for cerebellar development is proper morphology of Bergmann glia and abundance of Bergmann glia cells, both of which appeared to be adversely affected in *Fgfr1*/*Fgfr2* double knockout mice (Smith et al., 2012). Furthermore, *Fgfr1/Fgfr2* double knockout was confirmed in the BLBP+ glial stem cells of the cerebellar anlage, implicating the occurrence of both FGFR1 and FGFR2 in proliferative glial stem cells (Smith et al., 2012). Our present findings corroborate these data by revealing extensive *Fgfr1* expression in BLBP+ cells of the cerebellar anlage and rhombic lip, and the more-developed cerebellum of E14.5 and P0.5 mice, respectively. These findings further support the notion that *Fgfr1* is expressed in proliferative glial stem cells of both the embryonic and perinatal brain. Interestingly, migrating granule cells did not appear to express Fgfr1. In the *Fgfr1*/*Fgfr2* double mutants, granule cell migration was severely impeded, suggesting this portion of the phenotype was non-cell autonomous. It further implies that the granule cells are dependent upon *Fgfr1*/*Fgfr2* signaling in Bergmann Glia for migratory signals and guidance.

Astrocytes are glial cells responsible for many different functions throughout the postnatal nervous system, including neurotransmitter uptake and recycling, forming the blood–brain barrier, providing metabolic support, and providing structural support to the brain. One of the first roles of astrocytes in the brain is in guidance of axons across the midline to form the corpus callosum (Smith et al., 2006; Shu, Puche & Richards, 2003; Shu & Richards, 2001). GFAP+ cells of the glial wedge and indusium griseum serve as signaling centers of secreted ligands that guide the developing corpus callosum axonal projections. The indusium griseum astrocytes are born from radial glial stem cells in the telencephalic midline during mid-neurogenesis, well before cortical astrocytes are formed (Smith et al., 2006; Shu, Puche & Richards, 2003; Shu & Richards, 2001; Silver, Edwards & Levitt, 1993; Silver & Ogawa, 1983). These early born GFAP+ astrocytes undergo soma translocation from the glial wedge to the presumptive indusium griseum, with this behavior dependent upon expression of *Fgfr1* (Smith et al., 2006). Although previous studies have shown *Fgfr1* to be highly expressed in the midline during this time, accurate cellular resolution was absent, along with the knowledge of whether or not *Fgfr1* was also expressed in the migrating astrocytes or just in the GFAP+ radial glial precursors. Our present study of P0.5 perinatal mice reveals *Fgfr1* is, in fact, highly expressed not only in the radial glial cells of the glial wedge, but also in the astrocytes undergoing soma translocation to the indusium griseum. BLBP+ radial glia throughout the telencephalon that are undergoing soma translocation at P0.5 also express *Fgfr1*. Furthermore, NeuN+ cells of the indusium griseum did not appear to express *Fgfr1*, further suggesting the importance of *Fgfr1* expressing astrocytes in development of midline glial structures. These findings advance and support previous studies by providing evidence that *Fgfr1* is not only persistently expressed in both midline radial glial stem cells and their migrating astroglia progeny at P0.5, but it may also be required on a continuous basis for proper development and maintenance of midline structures. FGF8 signaling has also been implicated in the formation of midline glial structures (Stewart et al., 2016; Huffman, Garel & Rubenstein, 2004). More recently, Fgf8 signaling has been shown to regulate the remodeling of the midline glial cells in order to form the corpus callosum (Gobius et al., 2016). Interestingly, callosal axonal fibers in the dorsal, but not ventral, corpus callosum were GFP positive (Figs. 6B and 6E). These may represent the fibers of neurons in the anterior cingulate that express *Fgfr1* (Fig. 7). Previous studies found that the pioneer axons of the corpus callosum arise from the anterior cingulate (Rash & Richards, 2001). Thus, it is reasonable to postulate that FGFR1 signaling is also present within the axons that pioneer the anterior midline; however, *Synapsin Cre* mediated inactivation of *Fgfr1* did not result in axon guidance defects (Smith et al., 2006).

Here, we find *Fgfr1* expression in the hypothalamus, consistent with previous studies (Gonzalez et al., 1994). The postnatal hypothalamus is responsible for a multitude of functions regarding the maintenance of homeostasis, and more recently was identified as an area of postnatal neurogenesis that responds to both FGF2 and FGF10 signaling (Haan et al., 2013; Robins et al., 2013). Furthermore, FGF2 is implicated in regulating the proliferative abilities of the α-tanycytes lining the walls of the third ventricle, while having no effect on β-tanycytes (Robins et al., 2013). These data suggest FGFR1, the primary receptor for FGF2, may be present in the α-tanycytes along the walls of the third ventricle and not in the β-tanycytes of the ventricular floor, which were found to be non-proliferative in response to FGF2 (Robins et al., 2013). However, our findings indicate *Fgfr1* expression in cells featuring morphology consistent of tanycytes of both the walls and floor of the third ventricle, with only the cells along the wall immunopositive for BLBP. Furthermore, FGF10+ tanycytes along the floor of the third ventricle were shown to be neurogenic, but it is unknown whether FGF10 is signaling through FGFR1 in this area (Haan et al., 2013). Interestingly, FGF2 and FGFR1 signaling were implicated in appetitive behavior, with inhibitory effects upon glucose sensing neurons of the lateral hypothalamus (Li et al., 1996). One potential hypothesis is that FGFR1 signaling in hypothalamic tanycytes is altering neuronal function within the glucose sensing neurons.

## Conclusion

FGFR1 participates in a multitude of critical regulatory functions in the CNS in all stages of development. Understanding which cell types express this vital receptor is crucial to improving our understanding of prenatal and postnatal brain development. Here, we have described *Fgfr1* expression in both the embryonic and perinatal brains of *tgFgfr1-EGFP+* mice, which illuminate *Fgfr1* expressing cells via EGFP expression driven by a Fgfr1 promoter. By utilizing this model and simple immunostaining for EGFP and cell-specific markers, we have been able to accurately describe cell-type identification of *Fgfr1* expressing cells. Our findings of regional *Fgfr1* expression are consistent with previously published *in situ* hybridization data, but with the added advantage of cellular resolution. Presently, our data reveal *Fgfr1* expression in proliferative zones containing BLBP+ radial glial stem cells, such as the cortical and hippocampal VZ, and cerebellar anlage of E14.5 mice, in addition to DCX+ neuroblasts. Furthermore, our data reveal *Fgfr1* expression in proliferative zones containing BLBP+ cells of the anterior midline, hippocampus, cortex, hypothalamus, and cerebellum of P0.5 mice, in addition to the early formed GFAP+ astrocytes of the anterior midline. The results presented here may be utilized in future studies to further advance our understanding of FGFR1, its many roles throughout life, as well as its participation in neuropsychiatric disorders.

##  Supplemental Information

10.7717/peerj.3519/supp-1 Supplemental Information 1 Immunostaining of E14.5 control mice for BLBP and GFP in the telencephalon Immunostaining of E14.5 control mice for BLBP and GFP, with DAPI counterstaining (A–C), revealed little to no GFP fluorescence in the developing cortex (A), lateral pallial-subpallial boundary (B), and the LGE (C). Immunostaining of E14.5 control mice for Tbr2 and GFP, with DAPI counterstaining (D and E), revealed little to no GFP fluorescence in the anterior midline (D) or hippocampal primordium (E).

10.7717/peerj.3519/supp-2 Supplemental Information 2 Astrocytes of the glial wedge express *Fgfr1* Immunostaining for GFAP (A, C) and GFP (B, D) without DAPI counterstaining (as observed in Fig 6D) in P0.5 *tgFgfr1-EGFP+* mice. GFAP colocalizes with *Fgfr1* promoter-driven GFP. Arrowheads indicate examples of double stained cells. Scale bars = 50 µm.

## Additional Information and Declarations

### Data Availability

The following information was supplied regarding data availability: The raw data is included in the figures in the manuscript.
