# Supplementary figures and images for: ­Glial and stem cell expression of murine Fibroblast Growth Factor Receptor 1 in the embryonic and perinatal nervous system
Source: PeerJ. 2017 Jun 29;5:e3519. doi: 10.7717/peerj.3519 (PMC5493973; doi:10.7717/peerj.3519)

Control

**A** BLBP  
GFP  
DAPI

**B** BLBP  
GFP  
DAPI

**C** BLBP  
GFP  
DAPI

**D** Tbr2  
GFP  
DAPI

**E** Tbr2  
GFP  
DAPI

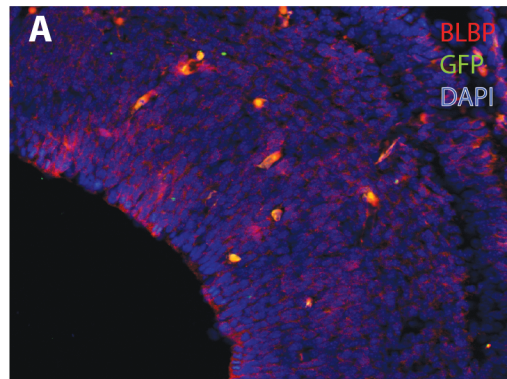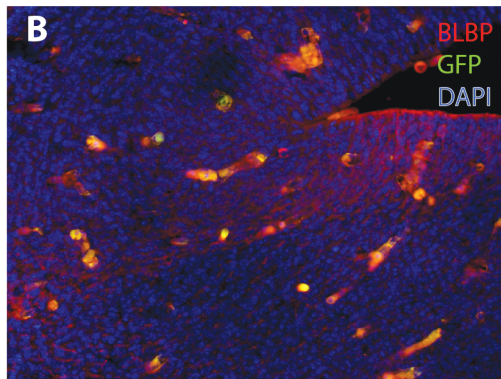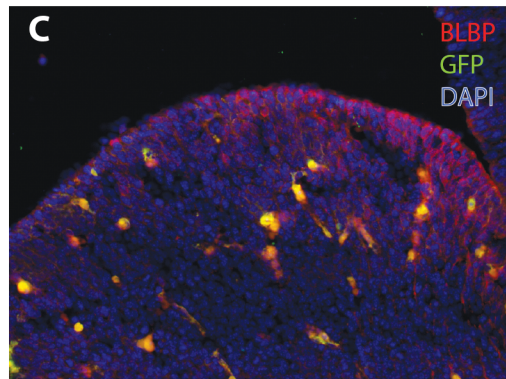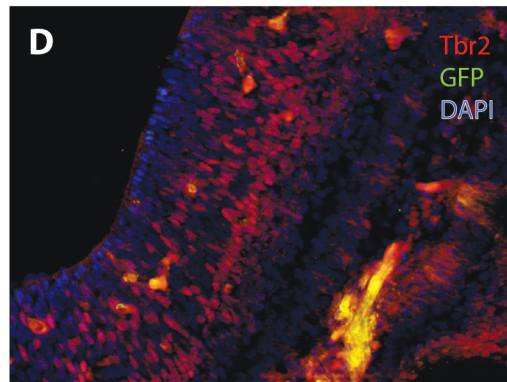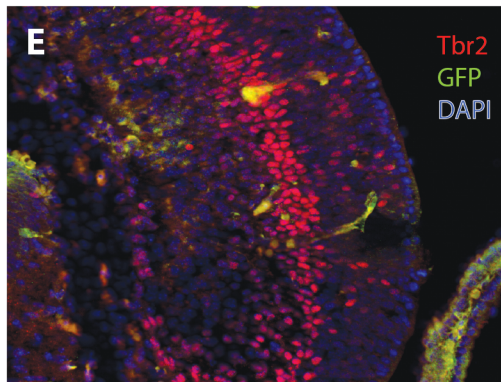

Supplement: Supplemental Information 1 — Immunostaining of E14.5 control mice for BLBP and GFP, with DAPI counterstaining (A–C), revealed little to no GFP fluorescence in the developing cortex (A), lateral pallial-subpallial boundary (B), and the LGE (C). Immunostaining of E14.5 control mice for Tbr2 and GFP, with DAPI counterstaining (D and E), revealed little to no GFP fluorescence in the anterior midline (D) or hippocampal primordium (E). [file peerj-05-3519-s001.pdf]

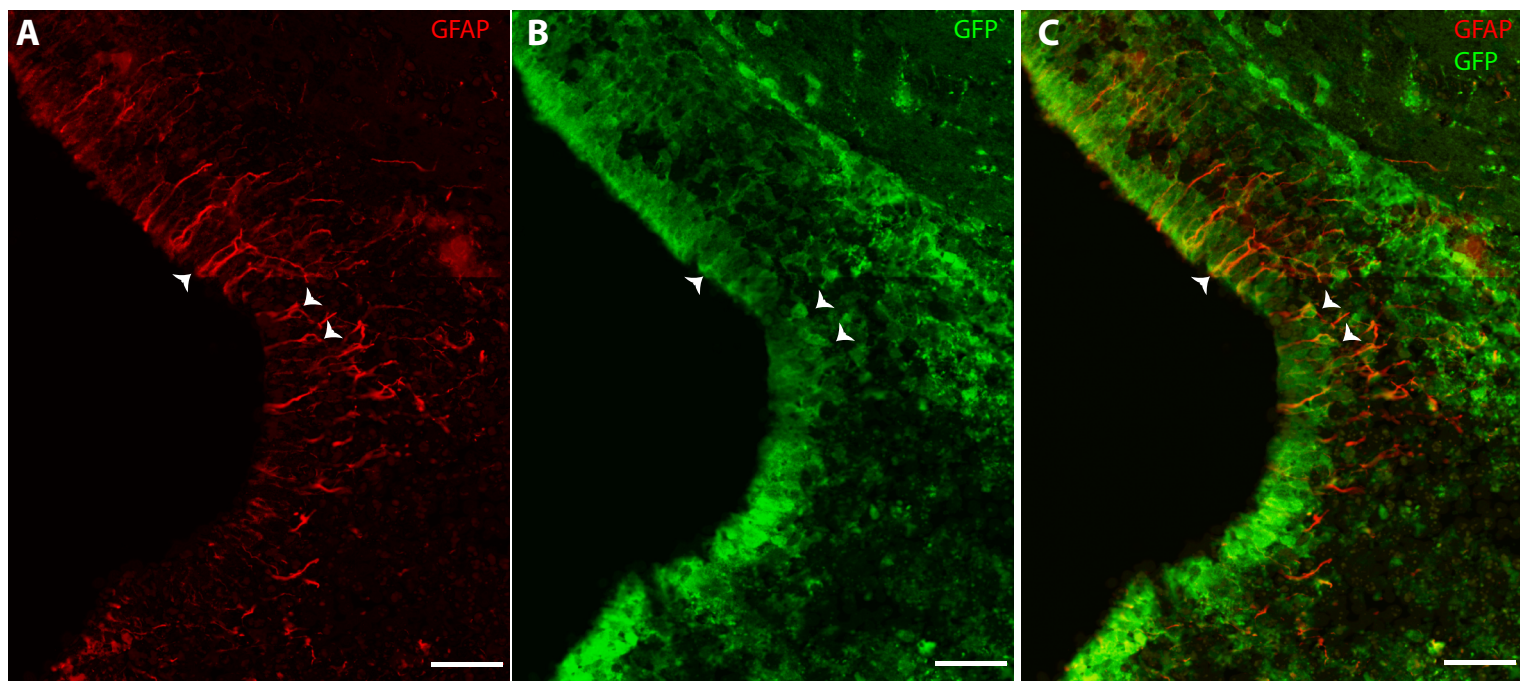

Supplement: Supplemental Information 2 — Immunostaining for GFAP (A, C) and GFP (B, D) without DAPI counterstaining (as observed in Fig 6D) in P0.5 tgFgfr1-EGFP+ mice. GFAP colocalizes with Fgfr1 promoter-driven GFP. Arrowheads indicate examples of double stained cells. Scale bars = 50 µm. [file peerj-05-3519-s002.pdf]
